# Supplementary material for: Interleukin 27, Similar to Interferons, Modulates Gene Expression of Tripartite Motif (TRIM) Family Members and Interferes with Mayaro Virus Replication in Human Macrophages
Source: Viruses. 2024 Jun 20;16(6):996. doi: 10.3390/v16060996 (PMC11209095; doi:10.3390/v16060996)
Supplement: Supplementary file 1 [file viruses-16-00996-s001.zip › Supplementary file S1.pdf]

Supplementary File S1. The gene symbol, subfamily symbol, and synonyms for each TRIM were reported in the literature [66–68]

Gene Symbol	Subfamily	Synonyms
TRIM1	I	MID2/FXY2
TRIM9	I	SPRING
TRIM18	I	MID1
TRIM36	I	Haprin
TRIM46	I	TRIFIC
TRIM67	I	
TRIM54	II	MURF3
TRIM55	II	MURF2
TRIM63	II	MURF1
TRIM42	III	
TRIML1	IV	
TRIM4	IV	
TRIM5α	IV	
TRIM6	IV	
TRIM7	IV	GNIP
TRIM10	IV	HERF1
TRIM11	IV	
TRIM15	IV	ZNFB7
TRIM17	IV	TERF
TRIM21	IV	RO52/SSA
TRIM22	IV	STAF-50
TRIM25	IV	EFP
TRIM26	IV	AFP
TRIM27	IV	RFP
TRIM34	IV	IFP1
TRIM35	IV	HLS5/MAIR
TRIM38	IV	RoRet
TRIM39	IV	TFP
TRIM41	IV	RINCK1
TRIM43	IV	
TRIM47	IV	GOA

TRIM48	IV	
TRIM49	IV	
TRIM50	IV	
TRIM53	IV	
TRIM58	IV	
TRIM60	IV	
TRIM62	IV	
TRIM64	IV	
TRIM65	IV	
TRIM68	IV	
TRIM69	IV	TRIF
TRIM72	IV	
TRIM75	IV	
TRIM8	V	GERP
TRIM19	V	PML
TRIM31	V	RING
TRIM40	V	
TRIM52	V	
TRIM56	V	
TRIM61	V	
TRIM73	V	
TRIM74	V	
TRIM24	VI	TIF1a
TRIM28	VI	TIF1b
TRIM33	VI	TIF1g
TRIM2	VII	NARF
TRIM3	VII	BERP
TRIM32	VII	BBS11
TRIM71	VII	
TRIM37	VIII	
TRIM23	IX	ARD1
TRIM45	X	
TRIM13	XI	LEU5
TRIM59	XI	

TRIM14	NO RING	
TRIM16	NO RING	
TRIM20	NO RING	MEFV
TRIM29	NO RING	
TRIM44	NO RING	
TRIM66	NO RING	
TRIM76	NO RING	SPRYD2
