# Supplementary figures and images for: Interleukin 27, Similar to Interferons, Modulates Gene Expression of Tripartite Motif (TRIM) Family Members and Interferes with Mayaro Virus Replication in Human Macrophages
Source: Viruses. 2024 Jun 20;16(6):996. doi: 10.3390/v16060996 (PMC11209095; doi:10.3390/v16060996)

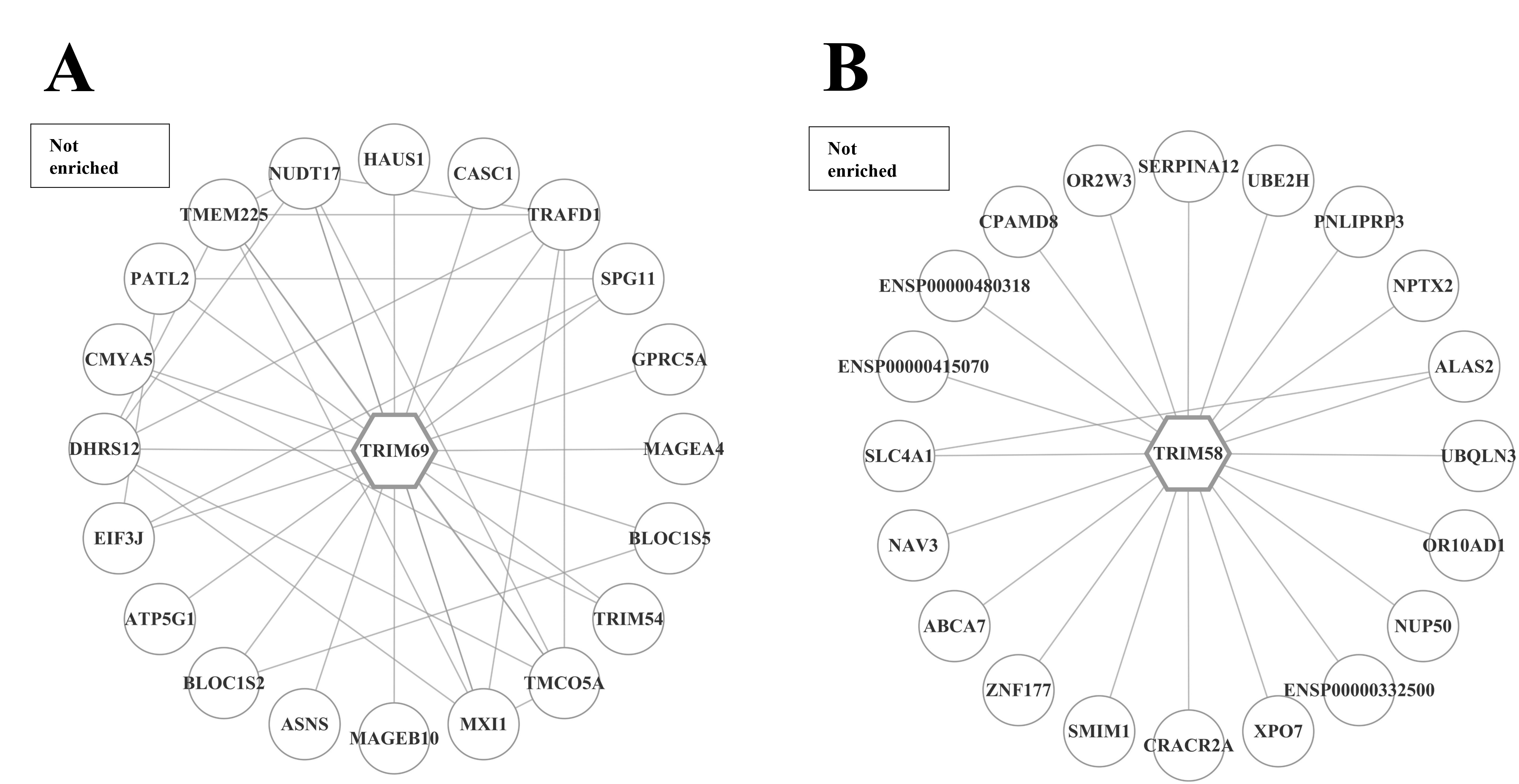

Supplement: Supplementary file 1 [file viruses-16-00996-s001.zip › Supplementary Figure S1.tif]
